# Pathology of Severe Malaria

**DOI:** 10.3390/pathogens12121389

**Published:** 2023-11-25

**Authors:** Julio Gallego-Delgado

**Affiliations:** 1 Department of Biological Sciences, Lehman College, The City University of New York, Bronx, New York, NY 10468, USA; julio.gallegodelgado@lehman.cuny.edu; Tel.: +1-347-577-4109; 2 Ph.D. Program in Biology, The Graduate Center, The City University of New York, New York, NY 10016, USA; 3 Ph.D. Program in Biochemistry, The Graduate Center, The City University of New York, New York, NY 10016, USA

Malaria, a devastating disease transmitted by mosquitoes, continues to plague many regions worldwide, affecting millions of lives annually. According to the latest World Malaria Report by the World Health Organization, there were 247 million new cases reported in 2021 [1]. Among those infected, a small but significant percentage develops severe malaria in one or more of its various forms, which poses a particularly grave threat [2]. In this Editorial, we examine recent multidisciplinary research that sheds light on the pathology of severe malaria, offering insights to deepen our understanding of this deadly disease. A central factor in the pathology of severe malaria is the immune response. The article “Characterization of Lymphocyte Subsets in Lymph Node and Spleen Sections in Fatal Pediatric Malaria” focused on understanding the lymphocyte subsets in pediatric malaria patients who died from two different clinical forms of severe malaria: severe anemia and cerebral malaria [3]. This research acts as a crucial piece of the puzzle in our understanding of immune dynamics and could pave the way for targeted therapies.

Cerebral malaria, which is the deadliest neurological manifestation of malaria, has been the main focus of severe malaria research for a long time. The article titled “Treatment Reducing Endothelial Activation Protects against Experimental Cerebral Malaria” highlights the critical importance of comprehending the pathophysiology of cerebral malaria [4]. The activation of endothelial cells in the brain microvasculature is a major contributor to the development of cerebral malaria [5]. Targeting this process could unlock new possibilities for potential therapies. While cerebral malaria has received much attention in severe malaria research, other equally devastating complications have been neglected for decades. One such complication that is of paramount significance is acute kidney injury (AKI) [6]. The article “Pathophysiology of Acute Kidney Injury in Malaria and Non-Malarial Febrile Illness: A Prospective Cohort Study” sheds light on this underexplored issue by examining markers of immune and endothelial activation in children in Uganda [7].

Understanding severe malaria, with its complex pathophysiology, requires a multifaceted approach, and animal models are an essential tool that help researchers in this endeavor [8]. The article, “Novel Experimental Mouse Model to Study Malaria-Associated Acute Kidney Injury,” exemplifies the pivotal role that these models play in advancing our comprehension of the disease [9]. The development of a new mouse model that mimics malaria-associated acute kidney injury is a significant breakthrough. This model enables researchers to study the intricate pathology of AKI in severe malaria. It provides a platform for testing potential treatments and interventions, discovering new biomarkers and ultimately accelerating the implementation of adjunctive therapies for this specific condition.

Severe malaria poses a significant global challenge, but its impact varies across regions. *P. falciparum*, one of the five species of *Plasmodium* that infect humans, causes most of the severe and fatal cases [10]. However, the article “A Retrospective Review on Severe Malaria in Colombia, 2007–2020” reveals that the situation is different in this region. The study shows that *P. vivax* is responsible for most of the severe malaria cases in Colombia, challenging the widely accepted belief that only *P. falciparum* causes severe malaria [11]. To tailor effective intervention strategies, it is crucial to understand the specific challenges and patterns of severe malaria in each context. Another article, “Analysis of Fifty Years of Severe Malaria Worldwide Research,” offers a comprehensive analysis that reflects the collective wisdom of the scientific community [12]. This study helps us to understand the evolution of our understanding of severe malaria from fifty years ago to the present day, highlighting progress and persisting challenges.

Although the ultimate goal of eradicating malaria is being pursued, this ancient disease—once known as the “king of diseases”—remains a major threat and is still among the top five causes of death in children [13]. The ultimate goal is to eradicate malaria, but it is impossible to set a specific timeline for this due to the complexity and difficulty involved. In fact, the WHO Strategic Advisory Group on Malaria Eradication has stated that “eradication by a specific date is not a promise we can make” [14]. It is our moral responsibility to reduce the number of children that succumb to malaria every year until we are able to eliminate the disease completely. It is crucial to make international efforts not only to eradicate malaria but also to improve treatment for severe malaria complications and decrease the number of deaths.

In conclusion, the pathology of severe malaria is a multifaceted puzzle, and each of these articles represents a piece of critical research that contributes to a broader understanding of this complex disease and paves the way for better diagnosis and treatments.

## References

[1] World Health Organization (2022). World Malaria Report 2022.

[2] Taylor C., Namaste S.M.L., Lowell J., Useem J., Ye Y. (2021). Estimating the Fraction of Severe Malaria among Malaria-Positive Children: Analysis of Household Surveys in 19 Malaria-Endemic Countries in Africa. Am. J. Trop. Med. Hyg..

[3] Mandala W.L., Ward S., Taylor T.E., Wassmer S.C. (2022). Characterization of Lymphocyte Subsets in Lymph Node and Spleen Sections in Fatal Pediatric Malaria. Pathogens.

[4] Mota S., Bensalel J., Park D.H., Gonzalez S., Rodriguez A., Gallego-Delgado J. (2022). Treatment Reducing Endothelial Activation Protects against Experimental Cerebral Malaria. Pathogens.

[5] Tunon-Ortiz A., Lamb T.J. (2019). Blood brain barrier disruption in cerebral malaria: Beyond endothelial cell activation. PLoS Pathog..

[6] Conroy A.L., Hawkes M., Elphinstone R.E., Morgan C., Hermann L., Barker K.R., Namasopo S., Opoka R.O., John C.C., Liles W.C. (2016). Acute Kidney Injury Is Common in Pediatric Severe Malaria and Is Associated with Increased Mortality. Open Forum Infect. Dis..

[7] Hawkes M.T., Leligdowicz A., Batte A., Situma G., Zhong K., Namasopo S., Opoka R.O., Kain K.C., Conroy A.L. (2022). Pathophysiology of Acute Kidney Injury in Malaria and Non-Malarial Febrile Illness: A Prospective Cohort Study. Pathogens.

[8] Mukherjee P., Roy S., Ghosh D., Nandi S.K. (2022). Role of animal models in biomedical research: A review. Lab. Anim. Res..

[9] Bensalel J., Roberts A., Hernandez K., Pina A., Prempeh W., Babalola B.V., Cannata P., Lazaro A., Gallego-Delgado J. (2023). Novel Experimental Mouse Model to Study Malaria-Associated Acute Kidney Injury. Pathogens.

[10] Walker I.S., Rogerson S.J. (2023). Pathogenicity and virulence of malaria: Sticky problems and tricky solutions. Virulence.

[11] Carmona-Fonseca J., Olivera M.J., Yasnot-Acosta M.F. (2022). A Retrospective Review on Severe Malaria in Colombia, 2007–2020. Pathogens.

[12] Garrido-Cardenas J.A., Gonzalez-Ceron L., Garcia-Maroto F., Cebrian-Carmona J., Manzano-Agugliaro F., Mesa-Valle C.M. (2023). Analysis of Fifty Years of Severe Malaria Worldwide Research. Pathogens.

[13] Collaborators G.B.D.U.-M. (2021). Global, regional, and national progress towards Sustainable Development Goal 3.2 for neonatal and child health: All-cause and cause-specific mortality findings from the Global Burden of Disease Study 2019. Lancet.

[14] Brew J., Pradhan M., Broerse J., Bassat Q. (2020). Researchers’ perceptions of malaria eradication: Findings from a mixed-methods analysis of a large online survey. Malar. J..

